# Maternal–Fetal Microtransfusions and HIV-1 Mother-to-Child Transmission in Malawi

**DOI:** 10.1371/journal.pmed.0030010

**Published:** 2005-11-22

**Authors:** Jesse J Kwiek, Victor Mwapasa, Danny A Milner, Alisa P Alker, William C Miller, Eyob Tadesse, Malcolm E Molyneux, Stephen J Rogerson, Steven R Meshnick

**Affiliations:** **1**Department of Epidemiology, University of North Carolina, Chapel Hill, North Carolina, United States of America; **2**Department of Community Health, College of Medicine, University of Malawi, Blantyre, Malawi; **3**Department of Pathology, Brigham and Women's Hospital, Boston, Massachusetts, United States of America; **4**Department of Obstetrics and Gynaecology, College of Medicine, University of Malawi, Blantyre, Malawi; **5**Malawi-Liverpool-Wellcome Trust Clinical Research Programme, College of Medicine, University of Malawi, Blantyre, Malawi; **6**Department of Medicine, University of Melbourne, Parkville, Victoria, Australia; Johns Hopkins Medical SchoolUnited States of America

## Abstract

**Background:**

Between 25% and 35% of infants born to HIV-infected mothers become HIV-1 infected. One potential route of mother-to-child transmission (MTCT) could be through a breakdown in the placental barrier (i.e., maternal–fetal microtransfusions).

**Methods and Findings:**

Placental alkaline phosphatase (PLAP) is a 130-kD maternal enzyme that cannot cross the intact placental barrier. We measured PLAP activity in umbilical vein serum as an indicator of maternal–fetal microtransfusion, and related this to the risk of HIV-1 MTCT. A case-cohort study was conducted of 149 women randomly selected from a cohort of HIV-1-infected pregnant Malawians; these women served as a reference group for 36 cases of in utero MTCT and 43 cases of intrapartum (IP) MTCT. Cord PLAP activity was measured with an immunocatalytic assay. Infant HIV status was determined by real-time PCR. The association between cord PLAP activity and HIV-1 MTCT was measured with logistic regression using generalized estimating equations. Among vaginal deliveries, PLAP was associated with IP MTCT (risk ratio, 2.25 per log_10_ ng/ml PLAP; 95% confidence interval, 0.95–5.32) but not in utero MTCT. In a multivariable model adjusted for HIV-1 RNA load, chorioamnionitis, and self-reported fever, the risk of IP MTCT almost tripled for every log_10_ increase in cord PLAP activity (risk ratio, 2.87; 95% confidence interval, 1.05–7.83).

**Conclusion:**

These results suggest that during vaginal deliveries, placental microtransfusions are a risk factor for IP HIV-1 MTCT. Future studies are needed to identify factors that increase the risk for microtransfusions in order to prevent IP HIV-1 MTCT.

## Abstract 2

Placental microtransfusions as measured by placental alkaline phosphatase levels in cord blood are a risk factor for mother-to-child transmission during vaginal deliveries.

## Introduction

By the end of 2004, sub-Saharan Africa was home to 13.3 million HIV-1-infected women, and in many sub-Saharan countries, the HIV-1 prevalence in antenatal women exceeded 20% [1]. In the absence of interventions such as antiretroviral drug prophylaxis, elective cesarean sections, and replacement feeding, between 25% and 35% of the children born to HIV-1-positive women themselves become infected [2]. Of the infant infections, approximately one-half occur during labor and delivery through an unknown mechanism [3].

One potential mechanism of HIV-1 mother-to-child transmission (MTCT) is through direct contact of infant mucosa with HIV-1-infected maternal blood, amniotic fluid, or cervical/vaginal secretions (the “all mucosal” mechanism) [4]. Evidence for the importance of this mechanism during intrapartum (IP) MTCT includes the observations that elective caesarean sections reduce HIV-1 MTCT [5], and that higher quantities of HIV-1 secreted into the birth canal are associated with increased HIV-1 MTCT [6,7]. On the other hand, both birth canal disinfection and emergency cesarean sections reduce birth canal exposure to HIV-1, but neither intervention significantly reduces HIV-1 MTCT [5,8–11].

In addition to the “all mucosal” hypothesis, HIV-1 MTCT could also occur via a breakdown in the maternal–fetal barrier followed by placental microtransfusions. Placental microtransfusions have previously been suggested as a route of HIV-1 transmission [12–15], and they are considered a plausible route of hepatitis B, C, and G vertical transmission [16–18]. Recently, an assay has been developed to quantify placental microtransfusions based on the detection of placental alkaline phosphatase (PLAP) activity in umbilical cord serum [13,15]. PLAP is a 130-kD glycoprotein synthesized from the eighth week of gestation until parturition, and its large molecular size is believed to preclude its passive diffusion across the placental barrier. Based on these characteristics and the observation that infants produce small amounts of PLAP compared to pregnant women, cord PLAP can be used as a surrogate marker of placental microtransfusions [13].

Although the cause of placental microtransfusions is unknown, they are hypothesized to arise during the first stage of labor, when uterine contractions intensify and membranes rupture [3,12,13]. In support of this hypothesis, cesarean sections performed before the onset of labor result in significantly less cord PLAP activity than both emergency cesarean deliveries and spontaneous vertex deliveries [13,15]. To build on these observations and to expand our knowledge of the biological mechanism of HIV-1 MTCT, we measured cord PLAP activity in Malawian mother–offspring pairs and evaluated placental microtransfusions as a risk factor for HIV-1 MTCT.

## Methods

### Participant Recruitment

This case-cohort study was derived from the Malaria and HIV in Pregnancy (MHP) prospective cohort study that was approved by the College of Medicine Research Committee at the University of Malawi and the Institutional Review Boards of the University of Michigan and the University of North Carolina at Chapel Hill. From December 2000 until March 2004, women in the Antenatal Ward at Queen Elizabeth Central Hospital in Blantyre, Malawi, were screened for eligibility to participate in a prospective cohort study designed to determine the association between malaria and HIV-1 MTCT. Women were ineligible for the study if they were in the active phase of labor, were participating in other research studies, lived outside the Blantyre district, were less than 15 y of age, were hypertensive, or had altered consciousness. Consenting women received HIV pre- and post-test counseling, and all HIV-1-infected women and their offspring received nevirapine according to the HIVNET 012 protocol [19]. The association between malaria and HIV-1 viral load in the MHP cohort has been described previously [20].

### Case-Cohort Design

We constructed a case cohort study from the parent MHP prospective cohort. With this study design, a rare disease assumption is unnecessary and the odds ratio provides a direct estimate of the risk ratio (RR) [21]. Between December 2000 and February 2003, 2,557 pregnant women were recruited in the MHP study, of whom 744 (29.1%) were HIV infected. After identifying all cases of in utero (IU) and IP MTCT in the parent cohort, we randomly selected 160 women, regardless of their HIV-1 transmission status, from the 744 HIV-1-positive women to serve as a reference cohort. Of these mother–offspring pairs, three were excluded because of multiple gestations, six were excluded because they delivered at home (therefore cord serum was unavailable), and two were excluded because the mother died during delivery; the remaining 149 HIV-infected women formed the reference cohort of the case-cohort study (Figure 1). All cases of IU MTCT were selected from women enrolled in the MHP study through February 2003, and all cases of IP MTCT were selected from women enrolled in the MHP study through June 2003. IP transmission cases selected from February to June 2003 did not differ from the other IP transmission cases in terms of age, peripheral HIV-1 RNA load, PLAP activity, hemoglobin concentration, placental malaria infection, or mode of delivery (data not shown). HIV-1 MTCT cases were considered IU transmissions if the infant was positive for HIV-1 DNA within 48 h of birth, and were considered IP transmissions (i.e., HIV-1 infection occurring at or around the time of delivery) if the infant was both negative for HIV-1 DNA within 48 h of birth and positive for HIV-1 DNA 6 wk after delivery [22].

**Figure 1:**
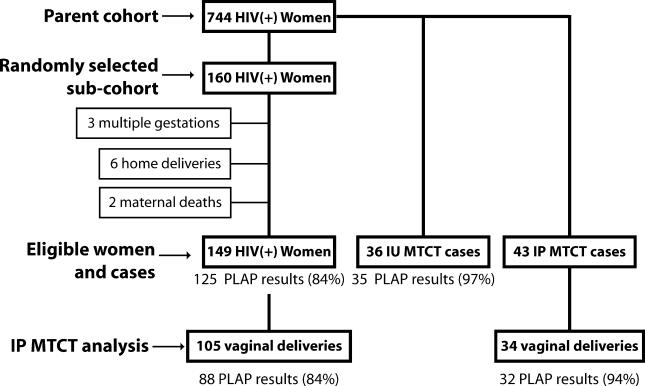
Case-Cohort Profile

### Laboratory/Pathology Testing

Chorioamnionitis was assessed by a board-certified pathologist (DM) according to the methods described in [23]. Peripheral malaria infection was assessed on thick blood films stained with field stain. Placental malaria was diagnosed from formalin-fixed placental biopsies as described in [24]. Maternal hemoglobin concentration was determined by HemoCue hemoglobinometer (HemoCue, Ängelholm, Sweden), and CD_4_-positive T cells were quantified by FACScan (Becton Dickinson, San Jose, California, United States).

### HIV-1 Testing

Within the MHP cohort, maternal HIV-1 status was determined concurrently with both the Determine HIV-1/2 Rapid Test (Abbott Laboratories, Abbott Park, Illinois, United States) and the SeroCard HIV-1/2 Rapid Test (Trinity Biotech, Bray, Ireland). HIV-1 RNA was quantified using Amplicor HIV-1 Monitor v1.5 (Roche Diagnostics, Branchburg, New Jersey, United States), with plasma HIV-1 RNA concentrations less than 400 copies/ml assigned a value of 400 copies/ml. Infants were considered HIV-1 infected based on the detection of HIV-1 DNA with a real-time PCR assay against the HIV-1 long terminal repeat [25]. Maternal HIV-1 proviral load was quantified according to the methods presented in [6].

### PLAP Assay

Immediately after delivery the umbilical cord was clamped and cut. A section of the umbilical cord 3 cm from its point of insertion into the placenta was washed with saline, the umbilical vein was located, and cord blood was aspirated with a large-bore needle. Serum was prepared from the blood and stored at −80 °C. PLAP was isolated from 100 μl of cord serum using an isoform-specific anti-PLAP antibody (clone B431, Biomeda, Foster City, California, United States), and its activity was measured with an immunocatalytic assay according to the methods of Hirano et al. [26] with the following modification: a fluorescent alkaline phosphatase substrate (5 μM fluorescein diphosphate, Molecular Probes, Eugene, Oregon, United States) was used, and its fluorescence was measured on a PerkinElmer (Wellesley, Massachusetts, United States) fluorimeter (excitation λ = 490 nm, 10-nm slit width; emission λ = 514 nm, 2.5-nm slit width, 515-nm cutoff filter). Cord PLAP concentration was determined by interpolation from a standard curve of purified human PLAP (Sigma, St. Louis, Missouri, United States). The standard curve was linear from 12 ng/ml to 3,250 ng/ml (*R* ^2^ = 0.992), with PLAP activity less than 12 ng/ml assigned a value of 12 ng/ml.

### Statistical Methods

IU and IP MTCT cases were analyzed as independent outcomes. We used generalized estimating equations with a logit link, binomial distribution, and an independent correlation structure to conduct bivariable and multivariable assessments of the relationship between maternal features and MTCT, while accounting for the lack of independence of the 23 MTCT cases included in the sub-cohort. To reflect the monotonically increasing risk of IP transmission as PLAP increased, log_10_ cord PLAP was coded as a continuous variable. In the multivariable models, prior to the outcome analysis, we assessed heterogeneity of the odds ratio by testing for interaction terms at α = 0.1. Subsequently, confounding was assessed by the backward elimination method [21] based on the change in the point estimate; variables that changed the estimate (RR) more than 10% were retained in the final model, with the exception of maternal HIV-1 viral load, which was included a priori. Variables assessed for interaction and potential confounding of HIV-1 MTCT prior to the outcome analysis include the following: HIV-1 RNA load, HIV-1 DNA load, CD4 T cell count < 200 cells/μl, chorioamnionitis (present or absent), episiotomy, self-reported fever in the week prior to enrollment, gestational age, placental malaria (any *Plasmodium falciparum*–infected erythrocytes on placental histology), and rupture of membranes more than 4 h prior to delivery. Owing to the correlation between HIV-1 RNA load, HIV-1 DNA load, and CD4 T cell count, the multivariable model of MTCT contained only one of these measures of HIV burden. Before the analysis of the association between maternal features and log_10_ cord PLAP, we eliminated duplicate values by removing the 23 cases included in the sub-cohort. The relationships between PLAP and dichotomous maternal factors were assessed with a two-tailed unpaired *t*-test (α = 0.05), and with continuous maternal factors the relationship was assessed with Pearson's correlation coefficient (α = 0.05). Statistical analysis was performed with S TATA v8.2 (StataCorp, College Station, Texas, United States).

## Results

We designed a case-cohort study to independently compare 36 IU and 43 IP MTCT cases to a sub-cohort of 149 HIV-positive mothers. The IU and IP MTCT cases were similar to the sub-cohort in terms of maternal age, infant birth weight, maternal hemoglobin concentration, gestational age, and gravidity (Table 1). The groups did not differ in the proportion of chorioamnionitis infections, nor in the proportion of peripheral or placental malaria infections. Of 205 deliveries, 149 (73%) were spontaneous vertex deliveries, 40 (20%) were emergency cesarean sections, seven (3%) were instrumental vaginal deliveries, five (2%) were elective cesarean sections, and four (2%) were breech deliveries; the mode of delivery did not differ significantly between study groups.

**Table 1:**
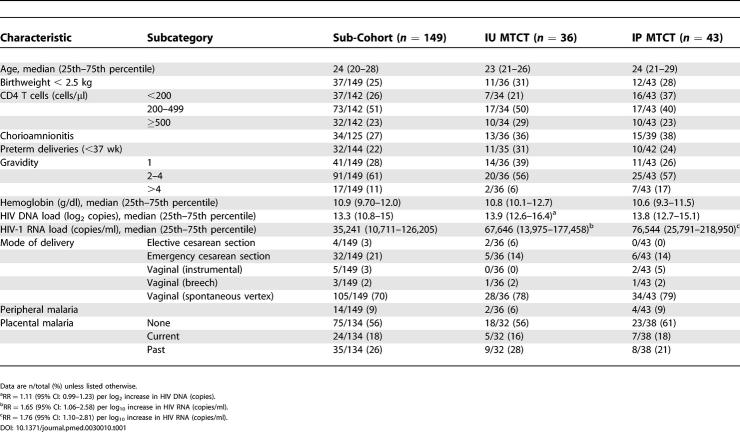
Enrollment Characteristics

As expected, HIV-1 RNA concentration was associated with MTCT. The median HIV-1 RNA load of the mother in IU MTCT cases was twice the median HIV-1 RNA load of the sub-cohort (67,646 copies/ml versus 35,241 copies/ml), and in a univariable regression, the risk of IU transmission increased 65% for every log_10_ increase in HIV RNA load (RR, 1.65; 95% confidence interval [CI], 1.06–2.58). Similarly, the median HIV-1 RNA load of the mother in IP MTCT cases was approximately twice the median RNA load of the sub-cohort (76,544 copies/ml versus 35,241 copies/ml), and the risk of IP MTCT increased 76% per log_10_ increase in HIV-1 RNA (RR, 1.76; 95% CI, 1.10–2.81). No statistically significant association between MTCT and either HIV DNA level or CD4 count was observed.

To validate PLAP as a marker for placental microtransfusions, we determined whether cord PLAP was associated with mode of delivery and gestational age. Cord serum was available for 177/205 (87%) of the enrolled mother–offspring pairs. Mean cord PLAP activity was lower in elective cesarean deliveries than in spontaneous vertex deliveries (1.43 log_10_ ng/ml versus 1.82 log_10_ ng/ml, *t =* −2.09, *p =* 0.039), and it was also lower in elective cesarean deliveries than in emergency cesarean sections (1.43 log_10_ ng/ml versus 1.81 log_10_ ng/ml, *t =* −1.78, *p =* 0.083). In addition, cord PLAP activity was directly correlated with gestational age (*n =* 174, correlation coefficient = 0.18, *p =* 0.017), and therefore preterm deliveries (<37 wk) had lower cord PLAP than term deliveries (37–40 wk) (1.68 log_10_ ng/ml versus 1.84 log_10_ ng/ml, *t =* 1.91, *p =* 0.058). In contrast, log_10_ cord PLAP was not associated with the following maternal variables: rupture of membranes more than 4 h prior to delivery (*n =* 163, *t =* 0.74, *p =* 0.46), placental malaria (*n =* 167, *t =* −0.12, *p =* 0.90), chorioamnionitis (*n =* 162, *t =* 0.85, *p =* 0.40), duration of labor (*n =* 177, correlation coefficient = −0.0001, *p =* 0.99), log_10_ HIV-1 RNA load (*n =* 170, correlation coefficient = 0.012, *p =* 0.88), or CD4 T cell count (*n =* 171, correlation coefficient = 0.12, *p =* 0.105). Therefore, the only maternal factors associated with cord PLAP were mode of delivery and gestational age.

The association between IP HIV MTCT and cord PLAP concentration was also measured. This association varied by the mode of delivery (*p =* 0.058), so prior to the analysis, the data were stratified by mode of delivery. In a univariable model of 119 spontaneous vaginal deliveries, elevated cord PLAP activity increased the risk of IP MTCT (RR, 2.25 per log_10_ ng/ml PLAP; 95% CI, 0.95–5.32); a bivariable model that included log_10_ HIV RNA load yielded a similar association (RR, 2.01; 95% CI, 0.84–4.79; *n =* 115). After adjusting the model for self-reported fever and chorioamnionitis, cord PLAP activity was significantly associated with IP MTCT (RR, 2.82 per log_10_ increase in cord PLAP; 95% CI, 1.04–7.67; *n =* 103; Table 2). The risk of IP MTCT associated with cord PLAP increased slightly if maternal CD4 T cell count or HIV-1 DNA load was substituted for HIV-1 RNA load, although the precision of the estimates varied (Table 2). For emergency cesarean section deliveries (*n =* 32), although the trend of the data suggests an inverse relationship, log_10_ PLAP was not associated with IP MTCT (RR, 0.32; 95% CI, 0.04–2.68). Small sample sizes precluded the analysis of an association between cord PLAP and HIV MTCT during instrumental vaginal, elective cesarean section, and breech deliveries. Thus, among vaginal deliveries, placental microtransfusions appear to be a risk factor for IP HIV-1 transmission.

**Table 2:**
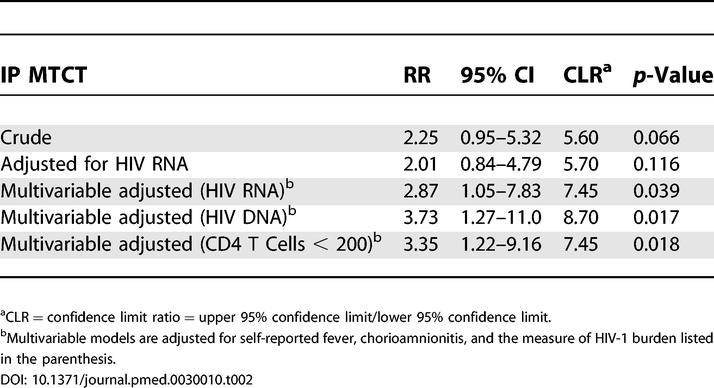
Risk of IP HIV-1 MTCT during Vaginal Delivery per log_10_ Increase in Cord PLAP

Finally, we analyzed the risk of IU MTCT from placental microtransfusions. In a univariable analysis of 159 mother–offspring pairs, log_10_ cord PLAP activity was not significantly associated with IU MTCT (RR, 0.54; 95% CI, 0.26–1.13). Inclusion of HIV-1 RNA load, preterm delivery, syphilis infection, chorioamnionitis, and placental malaria infection as covariates in the model did not change the magnitude of the relationship between cord PLAP and IU MTCT, which remained nonsignificant (data not shown). Thus, these data provide no evidence of an association between placental microtransfusions and IU HIV transmission.

## Discussion

In this case-cohort study of Malawian mother–offspring pairs, we tested the hypothesis that placental microtransfusions, as measured by cord PLAP activity, are an additional mechanism of HIV-1 MTCT. In support of this hypothesis, our data show that for every log_10_ increase in cord PLAP, the risk of IP transmission during vaginal deliveries almost tripled. Importantly, this increased risk of transmission remained after adjustment for HIV-1 RNA load, which is the most consistently reported risk factor for HIV-1 vertical transmission (reviewed in [27]).

In contrast to IP transmission during vaginal deliveries, placental microtransfusions were not significantly associated with IU HIV-1 transmission. The lack of association between IU MTCT and placental microtransfusions may result from the short half-life of PLAP in infants (~5 d) [13]; therefore, because PLAP activity is measured at the time of delivery, PLAP that passed into fetal circulation in the weeks prior to parturition might not persist until the time of delivery, and an association with IU MTCT, if it existed, would be missed.

Although a possible inverse relationship between PLAP and IP MTCT during emergency cesarean sections was observed, the small number of IP transmission cases in this stratum (*n =* 6) and the wide confidence intervals of this association (95% CI, 0.04–2.68) preclude a reliable conclusion.

The cord PLAP measurements in this study are consistent with previous studies in two ways. First, gestational age was directly correlated with cord PLAP activity, which is a result of the increase in maternal PLAP production over the course of a pregnancy [28]. Second, compared to both vaginal and emergency cesarean section deliveries, elective cesarean sections produced the smallest amount of microtransfusion [13,15]. Because only elective cesarean sections eliminate labor, the relationship between mode of delivery and cord PLAP activity has been attributed to a disruption of the placental barrier by labor and contractions. In support of this theory, Kaneda and colleagues reported a direct correlation between cord PLAP activity and prolonged labor (≥5 h) [13]. We did not observe a similar correlation among women who entered labor, and this could be explained in two ways: recording the duration of labor was not the primary concern of the study, so it is possible that our measurements were imprecise, or other features of labor such as the frequency and/or intensity of contractions may more strongly influence maternal–fetal transfusions.

Besides labor, placental infection or inflammation could also compromise the maternal–fetal barrier. A common source of placental pathology in sub-Saharan Africa is malaria, and although 17% of the women in this study had active placental malaria at the time of delivery, we detected no association between cord PLAP and placental malaria. A second potential source of placental inflammation is chorioamnionitis, which has been associated with HIV-1 MTCT [29]. Although 30% of the women in this study had chorioamnionitis, it also was not associated with cord PLAP. Based on these observations, there is no evidence from this study that placental malaria or chorioamnionitis increases placental microtransfusions.

The most probable confounding factor in this study is gestational age, which has been associated with both HIV transmission and maternal PLAP concentration. However, although the gestational age differed between the case-cohort groups, inclusion of gestational age in our IP transmission model did not change the risk estimate.

Two potential sources of uncertainty in our RR estimate are the use of PLAP as a marker of placental microtransfusions and the misclassification of HIV-1 transmission. Any measurement error in the PLAP assay (exposure) is likely to be nondifferential among the cases and the sub-cohort. Owing to the timing of infant blood collection (48 h and 6 wk after delivery), it is also possible that some of the HIV-1 transmission classified as IP was actually acquired late IU or during early breastfeeding [3]. This misclassification should be independent of PLAP activity, and therefore our RR estimate is most likely biased towards the null [21].

In this study investigating the role of maternal–fetal microtransfusions in HIV-1 MTCT, our data suggest that, independent of maternal HIV-1 viral load, placental microtransfusions during spontaneous vaginal deliveries increase the risk of IP HIV-1 MTCT. Future studies on the etiology of placental microtransfusions should provide greater insight into the mechanism of HIV-1 MTCT and suggest new strategies to prevent IP HIV-1 transmission in the developing world.

**Patient Summary**

**Background**

Without intervention, between 25% and 35% of the children born to HIV-positive mothers will themselves be infected. In about 50% of the cases, transmission from mother to child occurs during labor and delivery. Scientists don't yet understand how exactly this transmission happens. Even so, they have found that some treatments can prevent most of the mother-to-child transmission of HIV. The problem is that for many of the HIV-positive pregnant women in developing countries, these treatments are not available or not acceptable.

**Why Was This Study Done?**

The hope is that better understanding of mother-to-child transmission will lead to more effective, more available, and more acceptable treatments. One possible way children are infected is through placental microtransfusions, which are exchanges of small amounts of blood between the mother and the baby. Some degree of placental microtransfusion occurs in most pregnancies once labor starts, because the contractions cause small areas of rupture in the placenta, but the overall amount of blood exchange is likely to differ from delivery to delivery. Until recently, it was not possible to measure the extent of these microtransfusions for a particular delivery, but now scientists have developed an assay in umbilical cord blood that can do this. In this study, the researchers made use of this new assay to ask whether there is a link between the extent of placental microtransfusion and the likelihood of HIV transmission.

**What Did the Researchers Do and Find?**

They studied a group of mothers and children in Malawi. All of the mothers were HIV-positive, and some of them transmitted the virus to their children. This transmission occurred either during the pregnancy or around delivery, and the researchers knew the timing for each case. They also knew how the children were born: approximately three-quarters by vaginal delivery and one-fifth by emergency caesarean section. They determined the level of placental microtransfusion from the umbilical cord blood and then looked for correlations between HIV transmission and level of microtransfusion. They found no correlation for the cases where HIV was transmitted during pregnancy. For cases of transmission around delivery, however, higher levels of microtransfusion were associated with a higher risk of HIV transmission for vaginal deliveries.

**What Does This Mean?**

This suggests that better understanding of what causes microtransfusions might help researchers devise new strategies to prevent transmission. However, this will take some time. Because effective ways to prevent transmission already exist, the immediate goal must be to make them available to women in developing countries where transmission still occurs at high frequencies.

**Where Can I Find More Information Online?**

The following Web sites provide information on mother-to-child transmission of HIV.

World Health Organization pages:

http://www.who.int/reproductive-health/rtis/MTCT/

Joint United Nations Programme on HIV/AIDS pages:

http://www.unaids.org/Unaids/EN/In+focus/Topic+areas/ Mother-to-child+transmission.asp

Unversity of California at San Francisco HIV inSite pages:

http://hivinsite.ucsf.edu/InSite?page=kbr-07–02–03

Centers for Disease Control and Prevention page:

http://www.cdc.gov/nchstp/od/gap/pmtct/

Los Alamos HIV database:

http://hiv-web.lanl.gov/content/index

## Abbreviations

CI: confidence interval
IP: intrapartum
IU: in utero
MHP: Malaria and HIV in Pregnancy
MTCT: mother-to-child transmission
PLAP: placental alkaline phosphatase
RR: risk ratio
